# Vierordt’s Medical Diagnosis

**Published:** 1899-03

**Authors:** 


VIERORDT'S MEDICAL DIAGNOSIS. Fourth Edition, Revised. Medical Diagnosis. By DR. OSWALD VIERORDT, Professor of Medicine at the University of Heidelberg. Translated, with additions, from the fifth enlarged German edition, with the author's permission, by Francis H. Stuart, A. M., M. D. Handsome royal 8vo volume of 600 pages; 194 fine wood-cuts in text, many of them in colors. Philadelphia: W. B. Saunders. 1898.

This standard work on medical diagnosis has been revised throughout. The original plan of the author has not been changed. In every section we see the same painstaking, complete attention to detail. Every organ of the body is considered and the modern method of examination of the various physiological systems is presented clearly and concisely.

He takes in order the examination of the respiratory, circulatory, digestive and urinary apparatus. The chapter on examination of the nervous system includes over 100 pages. This has been thoroughly revised and brought up to date.

In the appendix is a consideration of laryngoscopy, rhinoscopy, otoscopy and ophthalmoscopy. The last section is a description of the bacteria which come under consideration in the diagnosis of internal diseases. As a book on diagnosis we know of none which is so complete and which is so helpful as Vierordt's.

